# The Effects of APOE and ABCA7 on Cognitive Function and Alzheimer’s Disease Risk in African Americans: A Focused Mini Review

**DOI:** 10.3389/fnhum.2019.00387

**Published:** 2019-11-05

**Authors:** Chelsie N. Berg, Neha Sinha, Mark A. Gluck

**Affiliations:** Center for Molecular and Behavioral Neuroscience, Rutgers University-Newark, Newark, NJ, United States

**Keywords:** African American (AA), APOE ε4, ABCA7, aerobic fitness, cognitive function, cognitive decline, Alzheimer’s disease

## Abstract

African Americans have double the prevalence of Alzheimer’s disease (AD), as compared to European Americans. However, the underlying causes of this health disparity are due to a multitude of environmental, lifestyle, and genetic factors that are not yet fully understood. Here, we review the effects of the two largest genetic risk factors for AD in African Americans: Apolipoprotein E (APOE) and ABCA7. We will describe the direct effects of genetic variation on neural correlates of cognitive function and report the indirect modulating effects of genetic variation on modifiable AD risk factors, such as aerobic fitness. As a means of integrating previous findings, we present a novel schematic diagram to illustrate the many factors that contribute to AD risk and impaired cognitive function in older African Americans. Finally, we discuss areas that require further inquiry, and stress the importance of racially diverse and representative study populations.

## Introduction

African Americans are at an elevated risk of cognitive decline and memory loss, with double the prevalence of Alzheimer’s disease (AD) as compared to European Americans (Logue et al., 2011; Barnes and Bennett, 2014; Alzheimer’s Association, 2019). The underlying causes of this health disparity are not sufficiently understood. Apolipoprotein E (APOE) and ABCA7, two genes involved in lipid metabolism, are the strongest heritable contributors to AD in African Americans (Reitz et al., 2013). However, the influence of genetic risk on environmental and behavioral risk factors, and their combined effects on AD biomarkers in African Americans, is yet to be determined. Furthermore, little is known about the neural substrates of cognition in older African Americans and how they relate to genetic risk factors for AD.

Here, we review recent work outlining two distinct ways genetic risk impacts AD biomarkers in African Americans. First, we examine the direct effects of genetic variation on neural correlates of cognitive function, such as activation and functional connectivity from functional magnetic resonance imaging (fMRI) studies. Second, we discuss the indirect effects of genetics on brain structure and function, *via* interaction with modifiable risk factors for AD, specifically aerobic fitness.

African Americans are at an increased risk of cardiovascular disease (Obisesan et al., 2012), which has been established as an important predictor for AD (Izquierdo-Porrera and Waldstein, 2002). Management or improvement of cardiovascular risk factors through increased aerobic fitness and exercise can reduce the risk for cognitive decline and dementia (Baumgart et al., 2015). Consistent with this, low levels of physical activity is one of the most prevalent risk factors for AD (Norton et al., 2014; Cass, 2017). In particular, African Americans have lower rates of physical activity as compared to European Americans (Gothe and Kendall, 2016; Benjamin et al., 2019). As such, aerobic fitness and exercise may be more viable modifiable factors to attenuate the risk for AD in African Americans.

It is important to delineate the difference between AD as determined by neuroimaging, biofluid biomarkers, or autopsy, as compared to the clinical diagnosis of Alzheimer’s and related dementias. However, to remain aligned with the terminology of the original cited works, throughout this review we refer to both instances as AD.

## Direct Effects of Genetics

### APOE

The APOE ε4 allele is one of the strongest genetic risk factors for AD (Potter and Wisniewski, 2012). APOE functions to regulate lipid metabolism in the brain by mediating the uptake of lipoproteins; in particular, it modulates the clearance of amyloid-β (Aβ; Di Paolo and Kim, 2011). Both dysfunctional cholesterol processing and Aβ aggregation have been implicated in AD pathogenesis (Schultz et al., 2017). In European Americans, the APOE ε4 allele has been associated with 2–3 times the risk of AD in heterozygotes and 12 times the risk in homozygotes (Michaelson, 2014). African Americans have a higher frequency of the APOE ε4 allele (Logue et al., 2011; Barnes and Bennett, 2014), and ε4 homozygosity is highly associated with AD in African-ancestry groups (Hendrie et al., 2014). However, the results are inconsistent for heterozygotic carriers (Farrer et al., 1997), with some studies suggesting that APOE ε4 may have less predictive impact on AD outcomes in African-ancestry populations, including African Americans (Rajabli et al., 2018). Despite the mixed nature of these findings, APOE ε4 has been associated with increased risk of late onset AD (LOAD) in African Americans (OR = 2.31; 95% increased risk; Reitz et al., 2013).

APOE ε4 has also been linked to episodic memory-related dysfunction in the medial temporal lobe (MTL; Bookheimer et al., 2000; Filippini et al., 2009; Dennis et al., 2010; Michaelson, 2014), one of the earliest brain regions impacted by the progression of AD. APOE ε4 genotype and amyloid-induced synaptic pathology have been related to accelerated rates of AD pathology within the MTL (Potter and Wisniewski, 2012), particularly in hippocampal sub-regions in both rodent and human models (Palmer and Good, 2011).

Pattern separation—the ability to independently represent and store similar experiences by reducing mnemonic interference (Leal and Yassa, 2018)—relies on MTL function. As such, one way to characterize decline into mild cognitive impairment (MCI) and AD is by a shift away from pattern separation towards pattern completion, which is mediated by dysfunctional hippocampal hyperactivity (Yassa et al., 2011b). Impaired mnemonic discrimination is associated with atypical hyperactivation in the dentate gyrus (DG) and CA3 hippocampal subfields (Dickerson et al., 2005; Yassa et al., 2011a,b; Reagh et al., 2017) in healthy older adults (Toner et al., 2009; Stark et al., 2013) and those with MCI (Yassa et al., 2010; Bakker et al., 2012, 2015; Tran et al., 2017).

Research examining the impact of APOE ε4 genotype on MTL function, *via* performance on a mnemonic discrimination task, has yielded mixed results in different racial populations with varying degrees of cognitive impairment. A study in MCI patients reported no differences in hippocampal hyperactivation or mnemonic discrimination based on APOE ε4 status (Tran et al., 2017). Conversely, AD patients that were homozygotic carriers of the APOE ε4 allele performed worse on challenging mnemonic discriminations (Wesnes et al., 2014). When examining spatial mnemonic discrimination across cognitively impaired and unimpaired older adults, impaired ε4 carriers performed worse than unimpaired carries and either group of non-carriers (Sheppard et al., 2016).

These previous studies were primarily conducted in European American cohorts and/or did not report the specific racial breakdown of their subject pools. In a population of cognitively healthy older African Americans, there were APOE ε4-related impairments in mnemonic discrimination, coincident with hyperactivity in the left DG/CA3 and the CA1. Although the overall effect of APOE ε4 on AD outcomes in African Americans remains unclear (Farrer et al., 1997; Tang et al., 2001; Hendrie et al., 2014; Rajabli et al., 2018), this result may suggest that APOE ε4-related hippocampal dysfunction can manifest in healthy older African Americans and may be an indicator of future disease status.

While APOE ε4 is associated with a moderately increased risk for progression from MCI to AD-type dementia (Elias-Sonnenschein et al., 2011), it may not alter the disease progression during the preclinical period (Bondi et al., 1999; Bunce et al., 2004). However, the effect of APOE ε4 in the preclinical phase may be contingent on other factors such as the level of amyloid aggregation (Mormino et al., 2014) and homozygotic vs. heterozygotic status (Caselli et al., 1999). Clinically normal carriers of APOE ε4 with high levels of amyloid aggregation experienced the highest levels of cognitive decline as compared to ε4 non-carriers and those with lower Aβ aggregation (Mormino et al., 2014). Cognitively healthy APOE ε4 homozygotic carriers also experienced memory decline earlier than heterozygotic carriers (Caselli et al., 1999).

### ABCA7

Outside of APOE, ABCA7 is the strongest genetic risk factor for AD in African Americans (Reitz et al., 2013). As a member of the super-family of adenosine triphosphate (ATP)-binding cassette (ABC) transporters, ABCA7 is another gene that regulates the homeostasis of phospholipids and cholesterol in the central nervous system and peripheral tissues. ABCA7 gene expression has been linked to AD *via* the dysregulation of lipid metabolism (Zhao et al., 2015; Aikawa et al., 2018).

ABCA7 single nucleotide polymorphism (SNP) rs115550680 is associated with the development of LOAD in African Americans with an effect size (OR = 1.79; 70%–80% increase in risk) that is comparable to that of APOE ε4 (Reitz et al., 2013). ABCA7 rs115550680 is hypothesized to contribute to AD in African Americans through amyloid precursor protein (APP) processing and the suppression of Aβ clearance (Cukier et al., 2016).

In cognitively healthy elderly subjects and MCI patients, cortical Aβ load is associated with disrupted functional connectivity within the MTL and impaired memory performance (Song et al., 2015). As such, Aβ plaques may play a key role in facilitating tauopathy in the MTL, and therefore lead to disrupted functional connectivity in the MTL circuitry. Hardy and Selkoe (2002) suggest that one of the functions of ABCA7 in AD may be Aβ facilitated tauopathy: as Aβ deposition accumulates in cortical regions within the default mode network (DMN), it may lead to concurrent accumulation of tau tangles in the MTL *via* reciprocal connections through the entorhinal cortex (EC) (Pooler et al., 2015). Hence, the cortico-MTL circuit may be the neural network underlying ABCA7 rs115550680-related AD pathology.

A recently published study examining the impact of ABCA7 rs115550680 genotype on the cortico-MTL network function in a group of cognitively healthy older African Americans found ABCA7-related dissociation in EC resting state functional connectivity (Sinha et al., 2019). Specifically, the risk variant was associated with increased functional connectivity between the EC and other MTL regions, including hippocampal subfields, coincident with decreased connectivity between the EC and medial prefrontal cortex (mPFC; Sinha et al., 2019). These findings suggest that for individuals with the risk ABCA7 rs115550680 genotype, impaired cortical connectivity leads to a more functionally isolated EC at rest, which translates into aberrant EC-MTL hyper-synchronization (Sinha et al., 2019).

While direct claims cannot be made about the exact mechanism underlying the aforementioned alterations in cortico-MTL network function, when considering the relevance of Aβ in ABCA7-related AD pathogenesis, these results may reflect the combined reinforcement between amyloid and tau pathology in the EC (Sinha et al., 2019). Thus, anomalous MTL functional connectivity may be an additional neural correlate of future cognitive decline in African Americans. This ABCA7 variant is monomorphic in European Americans (Reitz et al., 2013; Machiela and Chanock, 2015), and consequently, it does not confer any increased risk for AD in this group. However, recent studies of functional connectivity in MCI and AD patients have reported a similar disconnection of the MTL from other nodes of the DMN, particularly mPFC, but increased connectivity locally within the MTL, between EC and other subregions of the MTL (Das et al., 2013; Pasquini et al., 2015). As such, MTL network dysfunction may be a ubiquitously applicable AD biomarker for preclinical AD detection.

## Indirect Effects of Genetics

### The Interaction With Aerobic Fitness

Modifiable lifestyle factors, such as diet, exercise, and aerobic fitness, contribute to AD risk. In particular, aerobic fitness is one cardiovascular disease management method that has been associated with decreased levels of cognitive decline and reduced risk of AD in several previous studies (Colcombe and Kramer, 2003; Kramer et al., 2005, 2006). Aerobic activity has been found to aid in brain lipid homeostasis and in the reduction of Aβ deposit accumulation (Maesako et al., 2012; He et al., 2017; Houdebine et al., 2017). Recent work has also argued that increased levels of aerobic fitness can attenuate the adverse influence of AD-related polygenic vulnerability derived from genes implicated in lipid homeostasis, including APOE and ABCA7 (Schultz et al., 2017).

In addition to ABCA7 rs115550680 (reviewed under Direct Effects of Genetics), which has been identified as a genetic risk factor for AD in African Americans, another ABCA7 SNP (rs3764650) has been identified as a susceptibility locus for AD in European Americans (Hollingworth et al., 2011; Naj et al., 2011). ABCA7 rs3764650 has a lower effect size in African Americans (OR = 1.23), increasing AD risk by about 10%–20% (Reitz et al., 2013). However, this SNP has been found to influence overall ABCA7 expression (the conversion of DNA instructions into functional products and proteins), and, dysfunctional ABCA7 expression levels are associated with AD risk (Vasquez et al., 2013; Aikawa et al., 2018).

While the overall effects of ABCA7 rs3764650 on cognition seem to be minimal (Vivot et al., 2015; Andrews et al., 2016, 2017), it has been found to alter cognition in subgroups stratified on factors such as gender and disease progression. In healthy elderly, an association between rs3764650 and cognitive decline was found selectively in females (Nettiksimmons et al., 2016), and, in individuals with a final diagnosis of MCI or AD, this SNP was associated with increased rates of memory decline (Karch et al., 2012; Carrasquillo et al., 2015).

A study of healthy older African Americans found that ABCA7 rs3764650 modulates the association between aerobic fitness level (as measured by maximal oxygen consumption, VO_2_ max) and mnemonic flexibility—the ability to flexibly apply and recombine information from past learning—as measured by generalization following rule learning (Berg et al., 2019). In particular, for carriers of the non-risk genotype, higher levels of aerobic fitness were significantly associated with fewer generalization errors. Conversely, carriers of the risk genotype did not show any relationship between aerobic fitness and generalization. Successful mnemonic flexibility is known to depend on the integrity of the MTL (Myers et al., 2002, 2008), a major site of neuroplasticity that is sensitive to the effects of exercise and aerobic fitness (Cotman et al., 2007). The results of Berg et al. (2019) therefore imply that the ABCA7 risk genotype may attenuate the neuro-protective value of aerobic fitness in cognitively healthy older African Americans.

Analogous to this study, others have found that in European Americans, APOE ε4+ individuals did not receive the same benefits as APOE ε4− individuals from higher levels of aerobic fitness or following an exercise intervention, with fitness only reducing the risk for dementia in non-carriers (Podewils et al., 2005; Lautenschlager et al., 2008). On the contrary, some self-reported studies of physical activity found that the neuro-protective effects of fitness were exclusive to APOE ε4 carriers (Schuit et al., 2001; Rovio et al., 2005; Smith et al., 2011). Additionally, in African Americans the APOE ε4 genotype has been found to influence exercise-related upregulation of BDNF (brain-derived neurotrophic factor), a gene associated with neuroplasticity and hippocampal volume (Erickson et al., 2011); non-carriers of the ε4 allele exclusively experienced a significant increase in BDNF levels after 6 months of exercise, while carriers did not (Allard et al., 2017).

Research on the interactive effects of aerobic fitness and genetic risk for AD is still in the early stages, with the various studies containing methodological and racial differences in subject populations. Albeit equivocal, these results do provide evidence of the modulating effect of genetic variation on modifiable AD risk factors.

## Discussion

**Figure 1:**
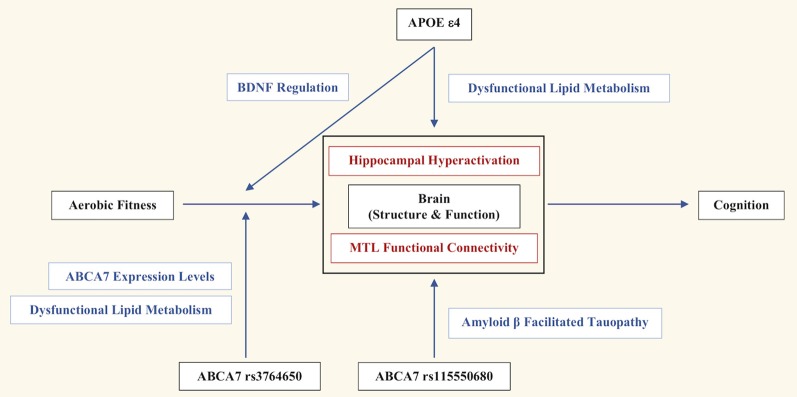
The genetic and lifestyle factors that contribute to Alzheimer’s disease (AD) risk and impaired cognitive function in African Americans. Overall, aerobic fitness influences brain structure and function, which then affects cognition. Apolipoprotein E (APOE) ε4 directly impacts brain structure and function *via* dysfunctional lipid metabolism, leading to aberrant hippocampal hyperactivation and therefore, impaired mnemonic discrimination of episodic memories. APOE ε4 indirectly influences the effects of aerobic exercise on hippocampal plasticity and volume through the regulation of BDNF. ABCA7 rs115550680 directly impacts the brain through amyloid-β (Aβ) facilitated tauopathy, which negatively influences medial temporal lobe (MTL) functional connectivity, and consequently, behavioral generalization. ABCA7 rs3764650 moderates the effects of aerobic fitness through dysfunctional lipid metabolism and ABCA7 expression, which indirectly impairs behavioral generalization.

Here, we reviewed research outlining the influence of genetic risk on MTL neural and cognitive function. We present a novel comprehensive outline of how genotypic variation may contribute to AD and impaired cognitive function (Figure 1). Overall, aerobic fitness influences neural structure and function, which then affects cognition. APOE ε4 directly impacts the brain *via* dysfunctional lipid metabolism, leading to aberrant hippocampal hyperactivation, and therefore, impaired mnemonic discrimination of episodic memories (Sinha et al., 2018). The indirect effects of APOE ε4 *via* fitness remain somewhat ambiguous, with some studies reporting aerobic fitness-related benefits only in APOE ε4− individuals (Podewils et al., 2005; Lautenschlager et al., 2008; Allard et al., 2017), while other studies report those benefits only in APOE ε4+ individuals (Schuit et al., 2001; Rovio et al., 2005; Smith et al., 2011). However, exercise-induced upregulation of BDNF, and its influence on hippocampal plasticity, may serve as a possible mechanism for the indirect influence of APOE ε4 (Allard et al., 2017).

Meanwhile, ABCA7 rs115550680 directly impacts the brain through Aβ facilitated tauopathy, which negatively influences MTL functional connectivity, and consequently, behavioral generalization (Sinha et al., 2019). Although ABCA7 rs3764650 is not a causative variant for AD in African Americans, and does not directly impact brain structure and function, it appears to confer indirect consequences on cognition and AD risk by moderating the effects of aerobic fitness through dysfunctional lipid metabolism and ABCA7 expression (Berg et al., 2019).

While the current schematic (Figure 1) of genetic influences on AD risk in African Americans is a first step, additional studies are needed to verify the molecular mechanisms underlying the link between genetic risk and pathogenic pathways; the potential contribution of brain lipid homeostasis in the MTL should be a focal point. It is also important to determine if ABCA7 and APOE have any common pathways mediating the effect on MTL structure and function. Furthermore, comprehensive single-cell type transcriptome analyses in human and mouse brains may be necessary to determine cell-specific contributions of ABCA7 risk variants to AD pathogenesis. For instance, ABCA7 rs115550680-related dysregulation of lipid metabolism may specifically target the neurons accelerating APP processing and Aβ production, while, ABCA7 rs3764650 may impact Aβ clearance by the microglia, known to play a pivotal role in mediating exercise-dependent enhancement of hippocampal neurogenesis (Vukovic et al., 2012).

Several studies have shown qualitative and quantitative differences in AD between African Americans and European Americans. One such study found racial differences in cerebrospinal fluid (CSF) and structural MRI biomarkers of AD in an elderly cohort; despite comparable CSF Aβ42 levels, white matter hyperintensity (WMH) volume, and hippocampal volume, the same degree of WMH had a greater influence on cognition in African Americans as compared to European Americans (Howell et al., 2017). Since WMH is a marker of vascular dysfunction, which African Americans experience at a higher rate than European Americans (Obisesan et al., 2012), these results may indicate that genes such as APOE and ABCA7, which regulate lipid metabolism, differentially affect African Americans. For example, the direct and indirect effects of ABCA7 have not been validated in other racial groups. ABCA7 rs115550680 is monomorphic on the non-risk minor “A” allele in European Americans (Reitz et al., 2013; Machiela and Chanock, 2015). As such, ABCA7 rs115550680 may confer AD risk selectively in African Americans, and, in conjunction with the indirect effects of ABCA7 rs3764650, may contribute to the higher incidence rate of dementia and AD in this population.

It is imperative that the studies presented here be replicated across diverse subject populations for a more representative and comprehensive understanding of AD progression and outcomes. At the same time, it will be crucial for future studies to examine race-specific AD biomarkers and consequences. Finally, researchers should explore the interplay between genetic variation and other modifiable lifestyle factors, such as diet and sleep patterns, to understand whether the benefits of potential interventions are similar for those with and without a genetic risk for dementia and AD.

## Author Contributions

CB and NS conducted background research. CB drafted the manuscript. NS and MG provided critical review of the manuscript.

## Conflict of Interest

The authors declare that the research was conducted in the absence of any commercial or financial relationships that could be construed as a potential conflict of interest.
